# Exposure to secondhand smoke among patients with asthma: a cross-sectional study

**DOI:** 10.31744/einstein_journal/2020AO4781

**Published:** 2020-01-23

**Authors:** Liranei Limoeiro Lima, Constança Margarida Sampaio Cruz, Andréia Guedes Oliva Fernandes, Gabriela Pimentel Pinheiro, Carolina de Souza-Machado, Valmar Bião Lima, Luane Marques de Mello, Álvaro Augusto Cruz

**Affiliations:** 1 Universidade Federal da BahiaSalvadorBABrazil Universidade Federal da Bahia , Salvador , BA , Brazil .; 2 Escola Bahiana de Medicina e Saúde PúblicaSalvadorBABrazil Escola Bahiana de Medicina e Saúde Pública , Salvador , BA , Brazil .; 3 Faculdade de Medicina de Ribeirão PretoUniversidade de São PauloRibeirão PretoSPBrazil Faculdade de Medicina de Ribeirão Preto , Universidade de São Paulo , Ribeirão Preto , SP , Brazil .

**Keywords:** Asthma, Tobacco smoke pollution/adverse effects, Environmental exposure, Quality of life, Health policy

## Abstract

**Objective:**

To estimate the frequency of secondhand smoke exposure among patients with asthma.

**Methods:**

A cross-sectional study of asthma patients and non-asthmatic controls using questionnaires to identify secondhand smoke exposure at home, school, work, and public places.

**Results:**

We studied 544 severe asthma patients, 452 mild/moderate asthma patients, and 454 non-asthmatic patients. Among severe patients, the mean age was 51.9 years, 444 (81.6%) were female, 74 (13.6%) were living with a smoker, 383 (71.9%) reported exposure in public spaces and, of the 242 (44.5%) who worked/ studied, 46 (19.1%) reported occupational exposure. Among those with mild/moderate asthma, the mean age was 36.8 years, 351 (77.7%) were female, 50 (11.1%) reported living with a smoker, 381 (84.9%) reported exposure in public settings and, of the 330 (73.0%) who worked/ studied, 58 (17.7%) reported occupational exposure. An association between secondhand smoke exposure and disease control was found among patients with mild/moderate asthma. Among those interviewed, 71% of severe asthma patients and 63% of mild/moderate asthma patients avoided certain places due to fear of secondhand smoke exposure.

**Conclusion:**

Secondhand smoke exposure is a situation frequently reported by a significant proportion of asthma patients. Individuals with asthma are exposed to this agent, which can hamper disease control, exacerbate symptoms and pose unacceptable limitations to their right to come and go in public settings.

## INTRODUCTION

Poor asthma control is a problem commonly faced by health professionals treating asthma patients. Even when receiving adequate therapy and specialized care, many do not achieve proper disease, control and require additional care measures to keep feeling well. ^1^ Uncontrolled asthma increases the need for hospital admissions and emergency visits, affecting patients’ quality of life and increasing costs to families and governments. ^2 - 4^ Moreover, it also decreases productivity, since it contributes to more school and work absences. ^5^

Multiple factors are involved in poor asthma control, such as low adherence to treatment and medical recommendations, inadequate use of inhalers, presence of multiple and co-morbid conditions, besides passive smoking − a major modifiable risk factor. ^3 , 6^

Smoking affects approximately 1.3 billion people globally and is associated with the deaths of more than 6 million people/year, of which 5 million result from active smoking, and 600,000 from secondhand smoking. ^2^ Several authors have pointed out the negative effects of tobacco use not only on the health of active smokers, but also those involuntarily exposed to the chemical substances present in secondhand smoke, spread in the air and later deposited on surfaces, polluting the environment. ^7 - 9^

Cumulative exposure to secondhand tobacco smoke can aggravate the damage to the respiratory system of asthma patients, leading to increased bronchial secretion, worse bronchial hyperresponsiveness (BHR), reduced lung function, more frequent exacerbations, and poorer response to drug therapy. ^3 , 10 , 11^ Some studies have reported more frequent respiratory infections ^12^ and new cases of asthma ^3 , 7 , 11^ among individuals who hang around smokers.

The Global Tobacco Surveillance System (GTSS) supports the fight against tobacco use, ^13^ contributing to reduce the prevalence of smoking around the globe. However, secondhand smoking continues to be a challenge. ^14^ According to data from the Surveillance System for Risk and Protective Factors for Chronic Diseases by Telephone Survey (VIGITEL - *Vigilância de Fatores de Risco e Proteção para Doenças Crônicas por Inquérito Telefônico* ) in 2013, Salvador (BA) was the capital with the lowest level of secondhand smoke exposure at home (7.5%) in Brazil, ^15^ and this level was even lower in 2014 (5.4%). ^16^ As for workplace exposure, surveys in 2013 and 2014 revealed a worrisome picture, with a 9.1% exposure to secondhand smoke. ^15 , 16^ In addition to secondhand smoking being more frequent than active smoking (5.1%), ^17^ these data are even more concerning, since there are no efficient methods to avoid cigarette smoke exposure in closed environments, and mechanisms are needed to protect those who do not smoke. Considering this is a modifiable risk factor, which may contribute to poor asthma control, we must understand the real conditions of this type of exposure among asthma patients at different levels of disease severity, to think of potential intervention strategies to minimize its negative impact on their disease. ^14^

## OBJECTIVE

To estimate the frequency and characteristics of secondhand smoke exposure in patients with asthma, and its association with disease control and severity.

## METHODS

### Study design

This is a cross-sectional study linked to the research line entitled Risk Factors, Biomarkers, and Endophenotypes of Severe Asthma, under the Bahia Asthma Control Program (ProAR - *Programa para o Controle da Asma na Bahia* ) of the *Universidade Federal da Bahia* , whose methodologies have been previously described. ^18^

### Recruitment and subjects

This study recruited patients with severe asthma, mild/moderate asthma (MMA) and non-asthmatics, adults (≥18 years), no sex distinction, living in Salvador (BA) or the surrounding metropolitan region, and users of the Brazilian Unified Health System (SUS - *Sistema Único de Saúde* ). Subjects with severe asthma were recruited from the ProAR cohort, provided they were on follow-up for at least 6 months on the day of the clinical evaluation. ^19^ They all had their diagnoses confirmed when admitted to the program, and disease severity was defined as per the criteria of the SAS/MS directive 1.012/2002 and the Global Initiative for Asthma (GINA), from 2002. ^19^ Mild/moderate asthma and non-asthmatics subjects were recruited from the community, through advertisements placed on primary care units, specialized outpatient clinics, public transportation, and other public places. The MMA diagnosis was confirmed as per the GINA criteria. ^20^ Non-asthmatics subjects identified after the clinical evaluation were also enrolled for verification of different exposure patterns.

We excluded subjects with comorbidities that prevented proper assessment of asthma control, such as congestive heart failure (CHF), myopathies, chronic lower respiratory disease, advanced neoplasm and pregnancy. ^18^

A call center was set up at the ProAR office to provide information on study details, and to check for fulfillment of the eligibility criteria. People indicated by ProAR patients were also contacted. For eligible candidates, a visit was scheduled for the signing of the Informed Consent Form (ICF) and starting the clinical evaluation and other study procedures.

### Data collection

This study reviewed data from 1,450 subjects (996 with asthma and 454 non-asthmatics), collected between 2013 and 2015.

Standard questionnaires were applied to collect information on socio-demographic characteristics and secondhand smoke exposure at home, at work/school, and in other public settings. Data on current and past smoking, medication use for asthma and other comorbidities, and alcohol and illicit drug abuse were also collected. At a second stage, interviewers applied an adapted questionnaire used by the Brazilian Institute of Geography and Statistics (IBGE - *Instituto Brasileiro de Geografia e Estatística* ) in the 2010 Census. ^21^ The Ministry of Health/VIGITEL questionnaire was also used to assess factors associated with the onset of chronic diseases. ^22^

Additionally, the Asthma Quality of Life Questionnaire (AQLQ) was used to check whether subjects stopped attending certain places to avoid exposure to secondhand cigarette smoke. ^23^

### Assessment of asthma control

Asthma control was assessed by the Asthma Control Questionnaire (ACQ), translated and validated in Brazil, ^24^ and scores ≥1.5 were indicative of uncontrolled asthma.

### Statistical analysis

Data were reviewed using the Software (SPSS), version 18.0. Descriptive statistics and comparisons are presented in tables. To measure association, we used the prevalence ratio (PR) and its respective 95% confidence intervals (95%CI).

### Ethical aspects

This study was approved by the Institutional Review Board of the *Maternidade Climério de Oliveira* of the *Faculdade de Medicina* at *Universidade Federal da Bahia* (opinion number 099/2009 and 095/2012).

## RESULTS

### Socio-demographic characteristics

The population studied was predominantly composed of women (81.8%) aged between 36 and 51 years. Significant differences relative to the variables presented were observed between the groups, except for self-reported skin color ( Table 1 ).

**Table 1:** Socio-demographic characteristics and self-reported current or past smoking of patients with severe asthma, mild to moderate asthma, and non-asthmatics

Characteristic	Severe asthma (n=544)	Mild/moderate asthma (n=452)	Non-asthmatic (n=454)	p value	Total (n=1,450)
Female sex	444 (81.6)	351 (77.7)	391 (86.1)	<0.01*	1,186 (81.8)
Age, years	51.85±13.47	36.84±12.79	44.07±12.55	<0.01 ^†^	44.74± 4.38
Family income, R$	1.186,32±1.272,47	1.412,78±1.073,09	1.290,45±1.458,23	<0.01*	1.287,71±1.284,78
Marital status				<0.01 ^†^	
Single	195 (35.8)	256 (56.6)	187 (41.2)		638 (44.0)
Married/stable relationship	234 (43.0)	163 (36.1)	189 (41.6)		586 (40.4)
Divorced	59 (10.8)	25 (5.5)	52 (11.5)		136 (9.4)
Widow (er)	56 (10.3)	8 (1.8)	26 (5.7)		90 (6.2)
Schooling level				<0.01*	
No schooling	37 (6.8)	3 (0.7)	8 (1.8)		48 (3.3)
Elementary school	127 (23.3)	25 (5.5)	51 (11.2)		203 (14.0)
Middleschool	129 (23.7)	62 (13.7)	106 (23.3)		297 (20.5)
High school	215 (39.5)	232 (51.3)	240 (52.9)		687 (47.4)
College/University	36 (6.6)	130 (28.8)	49 (10.8)		215 (14.8)
Skin color/race				0.68*	
Mixe drace	280 (51.5)	214 (47.3)	235 (51.8)		729 (50.3)
Black	215 (39.5)	195 (43.1)	180 (39.6)		590 (40.7)
Other ^‡^	49 (9.0)	43 (9.5)	39 (8.6)		131 (9.0)
Current smoking				<0.01 *	
No	539 (99.1)	434 (96.0)	417 (91.9)		1,390 (95.9)
Yes, daily	2 (0.4)	8 (1.8)	27 (5.9)		37 (2.5)
Yes, occasionally	3 (0.6)	10 (2.2)	10 (2.2)		23 (1.5)
Past smoking				<0.01*	
No	392 (72.7)	371 (86.1)	326 (77.8)		1,089 (75.1)
Yes	147 (27.3)	60 (13.9)	93 (22.2)		300 (20.7)

Results expressed by n (%) or mean±standard deviation. * χ ^2^ test (categorical variables); ^†^ Kruskal-Wallis test (continuous variables); ^‡^ white, indigenous, and yellow.

### Frequencies of current and past smoking

When reviewing data on smoking habits from the 2010 IBGE and the VIGITEL questionxnaires, lower rates of self-reported current smoking were found among patients with severe asthma (5 patients; 1%), followed by the MMA (18; 4.0%) and non-asthmatics (37; 8.1%) groups; with p<0.01. Among current smokers, 37 (2.5%) reported daily use of cigarettes and 23 (1.6%) occasional use ( Table 1 ).

Among non-smokers, self-reported past smoking was reported by 300 (20.7%) subjects, of which 147 (27.3%) had severe asthma, 60 (13.9%) had MMA and 93 (22.2%) were non-asthmatics ( Table 1 ).

### Characteristics of secondhand cigarette smoke exposure

When questioned on secondhand smoke exposure at home, 203 (14.0%) subjects reported to live with one or more smokers, of which 74 (13.6%) had severe asthma, 50 (11.1%) MMA, and 79 (17.4%) were non-asthmatics. Among severe asthma patients, 8.2% reported exposure to secondhand smoke at home, 19.1% at work/school, and 71.9% reported everyday exposure in public places ( Table 2 ).

**Table 2:** Characteristics of secondhand smoke exposure in different environments among patients with severe asthma, mild to moderate asthma, and non-asthmatics

		Severe asthma (n=544)		Non-asthmatic (n=454)	p value*		Total (n=1,450)	
How many smokers live in your residence?								
One	59 (10.8)			58 (12.8)		0.07		155 (10.7)
Two or more	15 (2.8)			21 (4.6)				48 (3.3)
Is smoking allowed in all the rooms in your residence?								
Yes	43 (7.9)			66 (14.5)		<0.01		161 (11.1)
How often do people smoke in your residence?								
Daily	37 (6.8)			48 (10.6)		<0.01		117 (8.1)
Weekly	8 (1.5)			3 (0.7)				27 (1.9)
Monthly	5 (0.9)			2 (0.4)				14 (1.0)
Less than once a month	2 (0.4)			16 (3.5)				23 (1.6)
For how long do you think you are exposed to cigarette smoke in your residence?								
Less than 1 hour/day	20 (3.7)			29 (6.4)		0.26		77 (5.3)
1to 4 hours/day	8 (1.5)			10 (2.2)				28 (1.9)
More than 4 hours/day	16 (3.0)			19 (4.2)				47 (3.2)
In the past 24 hours, for how long have you been exposed to cigarette smoke?								
Less than 1 hour/day	13 (2.4)			18 (4.0)		0.38		44 (3.0)
1 to 4 hours/day	8 (1.5)			11 (2.4)				29 (2.0)
More than 4 hours/day	12 (2.2)			29 (2.6)				29 (2.0)
Do you work or study outside your residence?								
Yes	242 (44.5)			288 (63.4)		<0.01		860 (59.3)
Are there any rules relative to smoking in closed environments at your workplace/school?								
Yes	164 (68.0)			204 (71.6)		0.61		602 (41.5)
How many people smoke around you at your workplace/school?								
One	20 (8.3)			25 (8.8)		0.75		69 (4.8)
Twoor more	28 (11.7)			40 (14.0)				104 (7.2)
For how long do you think you are exposed to cigarette smoke at your workplace/school?								
<1 hour/day	31 (12.9)			27 (9.5)		0.11		94 (6.5)
1 to 4 hours/day	3 (1.2)			11 (3.9)				28 (1.9)
More than 4 hours/day	12 (5.0)			17 (6.0)				37 (2.6)
In the past 24 hours, for how long have you been exposed to cigarette								
smoke at your workplace/school?								
<1 hour/day			17 (7.1)	21 (7.4)		0.22		51 (3.5)
1to 4 hours/day			2 (0.8)	2 (0.7)				9 (0.6)
More than4 hours/day			7 (2.9)	9 (3.2)				20 (1.4)
How often are you exposed to cigarette smoke in addition to the times at home								
and at school/work?								
<1 time/week	147 (27.6)			151 (33.6)		<0.01		461 (31.8)
>1 time/week	82 (15.4)			80 (17.8)				249 (17.2)
Daily	154 (28.9)			94 (20.9)				379 (26.1)
In the past 24 hours, for how long have you been exposed to cigarette smoke?								
<1 hour/day	139 (25.8)			91 (20.1)		0.45		329 (22.7)
1 to 4 hours/day	27 (5.0)			27 (6.0)				75 (5.2)
>4 hours/day	23 (4.3)			18 (4.0)				60 (4.1)

Results expressed by n (%). * χ ^2^ test.

When assessing exposure at work/school, we found that a large part of the subjects with severe asthma (55.5%) did not take part in any out-of-home activities. However, among those who did study or work, 173 (12.0%) reported exposure to passive smoking due to the presence of one or more smokers in their workplace or school.

There was no significant difference between the groups, however, the frequencies of this type of exposure reported by subjects were strikingly high (20.0% for severe asthma, 18.5% for MMA, and 22.8% for non-asthmatics). Furthermore, 58.5% said there were no rules prohibiting smoking at their workplace/school ( Table 2 ). We also found that exposure to secondhand smoke in public places was fairly frequent, as reported by 71.9% of subjects with severe asthma, 84.9% of those with MMA, and 72.3% of non-asthmatics subjects ( Table 2 ).

### Assessment of asthma control

When reviewing data on secondhand smoke exposure and asthma control ACQ, we found a higher rate of controlled asthma among asthma patients not exposed to secondhand smoke when compared to those exposed, both for MMA and severe asthma. However, an association was found between secondhand cigarette smoke exposure and asthma control only in subjects with MMA (PR= 2.04; 95%CI: 1.27-3.30) ( Table 3 ).

**Table 3:** Association between exposure to secondhand smoke and asthma control, by disease severity

Exposure	Severe asthma (n=544)			Mild/moderate asthma (n=452)		
	
	Controlled (n=352)	Uncontrolled (n=192)	PR (95%CI)	Controlled (n=386)	Uncontrolled (n=66)	PR (95%CI)
Present	56 (15.9)	33 (17.2)*	0.91	52 (13.5)	18 (27.3)*	2.04
			(0.57-1.46)			(1.27;3.30)
Absent	296 (84.1)	159 (82.8)*	p=0.7	334 (86.5)	48 (72.7)*	p<0.01

Results expressed by n (%). * Asthma Control Questionnaire ≥1.5 (uncontrolled asthma).

PR: prevalenceratio; 95%CI: 95% confidenceinterval.

When determining the percentage of subjects with asthma who avoided certain places due to fear of exposure to secondhand smoke, according to AQLQ data, we observed that most asthma patients had their social lives limited due to fear of exposure. Subjects with severe asthma reported avoiding certain places for this reason 18% more frequently than those with MMA (PR=1.18; 95%CI: 1.04-1.35) ( Table 4 ).

**Table 4:** Percentage of subjects with mild, moderate and severe asthma avoiding certain places due to fear of exposure to secondhand smoke

	Severe asthma (n=544)	Mild/moderate asthma (n=452)
Avoided	386 (71.0)	285 (63.1)
Did not avoid	158 (29.0)	167 (36.9)

Results expressed by n (%). Prevalence ratio (95% confidence interval) = 1.18 (1.04-1.35).

## DISCUSSION

This study brought relevant information about secondhand or passive smoking, a situation which, although known for a long time, remains difficult to approach. Our results showed that, among subjects with severe asthma, past smoking and secondhand smoke exposure were frequently reported. This group also had a higher rate of subjects unaware of regulatory standards for smoking in closed places, and a higher rate of secondhand smoke exposure in public places. The study also found a higher incidence of uncontrolled asthma among those exposed to secondhand cigarette smoke, particularly in the MMA group, as well as more frequent reports of avoiding certain places due to fear of exposure among patients with severe asthma.

As for socio-demographic characteristics, in the severe asthma group, there was a higher proportion of women and subjects who did not work or study, higher mean age and lower schooling level. This profile has also been found in a similar study. ^5^

Subjects who did not work were more frequent among severe asthma patients, which could be due to the higher severity and poor control of symptoms, as well as more frequent emergency visits and hospital admissions, contributing to more work/school absences. ^5 , 25^

Lower schooling levels was also found by other authors. ^26^ According to the latest Brazilian census, 50.2% of the population over 10 years old has never attended school or has incomplete middle education, which is in line with the profile of subjects in our study and other similar studies. ^27^ The schooling level must be considered when planning health education activities, since it affects the understanding by subjects of the questionnaires and interventions, and may give rise to data collection bias.

This study found a lower rate of current smoking among asthma patients, which could be due to limitations in the methodology employed (self-report). ^3 , 10^ A study in São Paulo also found a lower rate of current (3%) and secondhand (17%) smokers among asthmatics, and the authors discuss the importance of using straightforward techniques to detect smoking biomarkers, such as urinary cotinine. ^14^ A study conducted by our group aiming to compare self-reported active smoking and urinary cotinine concentrations revealed an omission rate of 5% in severe asthma and 4.8% in MMA patients, which emphasizes the importance of using straightforward measures in situations where tobacco use monitoring is relevant for disease management. ^28^

Exposure to secondhand cigarette smoke was reported by a significant part of the subjects in this study, both asthmatics and non-asthmatics. Despite the rate of current smoking being relatively low among asthmatics, we found that they remained exposed to secondhand smoke at home, including those with severe asthma. However, non-asthmatics subjects were the ones that most frequently reported living with smoker, freedom to smoke cigarettes in any room in the residence, and exposure to cigarette smoke at home. Although these people may not experience any immediate discomfort related cigarette smoke exposure, data from different studies have shown a higher risk of onset of asthma among those living with smokers. ^3 , 7 , 11^ This fact must be kept in mind when providing health counseling to both smokers and those living with a smoker. This approach is even more important when managing asthma, since secondhand smoke exposure can be related, among other factors, to failure of treatment response. ^3^

The permission to smoke in any room in a residence, irrespective of disease severity, was reported by nearly 10% of severe asthma and 13.3% of MMA subjects. This finding suggests mis information by family members and/or patients themselves, emphasizing the importance of health education actions in the monitoring of asthmatic patients. Educating on the risks is part of the process of empowering patients and family members, encouraging them to take an active role in their treatment and clinical improvement. Current smoking and secondhand smoke exposure lead to poorer response to treatment with corticosteroids and poorer asthma control, ^3 , 29^ in addition to decline in pulmonary function, which is more marked among smokers with asthma than non-asthmatic smokers, ^30^ a fact that can also be seen in children of smoking parents. ^31^

Federal law 12.546/2011, which bans smoking in closed places across the country, ^32^ and presidential decree 8.262/2014 ^33^ set forth measures to promote smoke-free environments. Nevertheless, 30% of subjects who worked/studied said they were unaware of any standards banning smoking in closed places, which points to the need for communicating this information and implementing proper surveillance.

A study with students found a high rate of subjects exposed to secondhand cigarette in public places (62.2% in Porto Alegre and 53.6% in Florianópolis). ^31^ These data are similar to those observed in our study, which identified a daily frequency of secondhand smoke exposure in public places, reported by 28.9% of patients with severe asthma and 29.0% of those with MMA. The lack of restrictions on smoking in public places may affect those with chronic respiratory diseases, ^22^ which reiterates the need for effective public policies to restrict this exposure, even if outdoors. ^16^

This study found exposure to secondhand cigarette smoke within the past 24 hours in a considerable number of subjects, particularly among those with severe asthma, which suggests frequent and current contact. It is possible that asthma patients are more sensitive and, therefore, can better notice the presence of smoke in the air and more frequently avoid it; however, the current study methodology did not allow for this type of assessment.

When assessing the relation between secondhand smoke exposure and asthma control, we found that patients with MMA have a 2.04 higher chance of having uncontrolled asthma upon contact with secondhand cigarette smoke than patients with severe asthma, which suggests that poor symptom control among patients with severe asthma may be linked with biological characteristics of the disease, rather than external factors, which may not be the case in MMA. Different studies have shown an association between exposure and worsening of airway inflammation and BHR, more frequent exacerbations, impaired quality of life, poor disease control and higher disease severity. ^3 , 10 , 11^ King et al., in a cross-sectional cohort study in 14 countries, found a significantly higher rate of secondhand smoke exposure in different places, and discussed the need for professional interventions. ^34^ Another study showed that smoking significantly interferes in increasing the dose of inhaled corticosteroids used by asthmatics and in disease control. ^35^ The percentage of people with asthma exposed to secondhand cigarette smoke in this study was significant, which must raise a red flag to professionals treating asthma on the importance of instructing patients to avoid this type of exposure, and ask active smokers nearby not to smoke in their presence, ^3 , 14^ due to the risk of symptom exacerbation and uncontrolled disease.

The consequences of this exposure to the lives of asthma patients encompass more than just the clinical repercussions. The quality of life of these people is affected not just during exacerbations, when they require more frequent emergency visits and hospital admissions. The answers to the AQLQ questionnaire showed that approximately 68% of all asthma patients studied admitted to avoiding certain places due to fear of exposure to secondhand cigarette smoke, and this behavior was significantly more frequent among severe asthma patients. This shows that these people are also affected on a day to day basis, even when the disease is better controlled, since their mobility must be restricted to avoid the consequences of secondhand smoke exposure.

## CONCLUSION

This study identified that one-third of asthma patients (with both mild/moderate and severe asthma) are frequently exposed to secondhand cigarette smoke at home, work/school and/or public places. More than two-thirds of patients with severe asthma reported avoiding places with higher chances of exposure due to fear of exacerbations and their consequences, which is unacceptable. This exposure is also associated with poor disease control in patients with mild/moderate asthma. New studies on this subject must be encouraged to confirm these results in other regions and situations. This information should inspire new interventions targeting everyday clinical practice and public policy making, with the view to benefit asthma patients.
