# Supplementary material for: Exposure to secondhand smoke among patients with asthma: a cross-sectional study
Source: Einstein (Sao Paulo). 2020 Jan 23;18:eAO4781. doi: 10.31744/einstein_journal/2020AO4781 (PMC6986455; doi:10.31744/einstein_journal/2020AO4781)
Supplement: Translation

# Translation: Exposição à fumaça secundária do cigarro entre pacientes com asma: estudo transversal

**Authors:** Liranei Limoeiro Lima, Constança Margarida Sampaio Cruz, Andréia Guedes Oliva Fernandes, Gabriela Pimentel Pinheiro, Carolina de Souza-Machado, Valmar Bião Lima, Luane Marques de Mello, Álvaro Augusto Cruz

**Affiliations:** 1 Brasil Universidade Federal da Bahia, Salvador, BA, Brasil .; 2 Brasil Escola Bahiana de Medicina e Saúde Pública, Salvador, BA, Brasil .; 3 Brasil Faculdade de Medicina de Ribeirão Preto, Universidade de São Paulo, Ribeirão Preto, SP, Brasil .

**Keywords:** Asma, Poluição por fumaça de tabaco/efeitos adversos, Exposição ambiental, Qualidade de vida, Política de saúde

## Abstract

**Objetivo:**

Estimar a frequência de exposição à fumaça secundária do cigarro entre pacientes com asma.

**Métodos:**

Estudo transversal, que avaliou pacientes com asma e controles sem asma, por meio de questionários, para identificar a exposição secundária à fumaça do cigarro no ambiente domiciliar, escolar, no trabalho e em ambientes públicos.

**Resultados:**

Estudamos 544 asmáticos graves, 452 com asma leve/moderada e 454 sem asma. Entre os asmáticos graves, a média de idade foi de 51,9 anos, 444 (81,6%) eram do sexo feminino, 74 (13,6%) tinham fumantes em sua residência, 383 (71,9%) relataram exposição em ambientes públicos e, dos 242 (44,5%) que trabalhavam e/ou estudavam, 46 (19,1%) admitiram exposição ocupacional. Entre asmáticos leves/moderados, a média de idade foi de 36,8 anos, 351 (77,7%) eram do sexo feminino, 50 (11,1%) afirmaram haver tabagistas em sua residência, 381(84,9%) relataram exposição em ambientes públicos e, dos 330 (73,0%) que trabalhavam e/ou estudavam, 58 (17,7%) referiram exposição ocupacional. Encontrou-se associação entre exposição à fumaça secundária do cigarro e controle da doença entre pacientes com asma leve/moderada. Entre os entrevistados, 71% dos pacientes asmáticos graves e 63% daqueles com asma leve/moderada relataram evitar frequentar certos ambientes pelo receio da exposição à fumaça secundária do cigarro − relato mais associado aos pacientes com asma grave.

**Conclusão:**

A exposição secundária à fumaça do cigarro é uma situação frequente e relatada por uma proporção significativa de asmáticos. Indivíduos com asma encontram-se expostos a este agente, que pode dificultar o controle da doença, exacerbar sintomas e lhes impor limitação inaceitável ao direito de ir e vir em ambientes públicos.

## INTRODUÇÃO

A falta de controle da asma é um problema comumente enfrentado pelos profissionais de saúde que tratam pacientes asmáticos. Mesmo submetidos a esquemas terapêuticos adequados e acompanhamento especializado, muitos não conseguem atingir a meta de controle da doença, necessitando de mais cuidados para se manterem bem. ^1^ A asma não controlada aumenta a necessidade de hospitalizações e visitas às emergências, comprometendo a qualidade de vida do paciente, e elevando custos para a família e o estado. ^2 - 4^ Além disto, também prejudica a produtividade do indivíduo, à medida que contribui para o absenteísmo escolar e laboral. ^5^

Vários fatores estão envolvidos na falta de controle da asma, como baixa adesão ao tratamento e às recomendações médicas, inadequação no uso de inaladores, presença de co- e multimorbidades, além do tabagismo passivo−importante fator de risco modificável. ^3 , 6^

O tabagismo atinge cerca de 1,3 bilhão de usuários em todo o mundo e está associado à morte de mais de 6 milhões de pessoas/ano, sendo cerca de 5 milhões decorrentes do tabagismo ativo, e 600 mil da exposição passiva à fumaça do cigarro. ^2^ Vários autores chamam a atenção para os prejuízos do tabaco para a saúde não apenas do fumante ativo, mas também para aquele que, involuntariamente, é exposto às substâncias químicas presentes na fumaça secundária, dispersa no ar durante sua combustão que, posteriormente, deposita-se em superfícies, gerando poluição ambiental. ^7 - 9^

A exposição à fumaça secundária do tabaco de forma cumulativa pode agravar danos ao sistema respiratório do asmático, resultando em aumento da secreção brônquica, piora da hiperresponsividade brônquica (HRB), função pulmonar reduzida, exacerbações mais frequentes e prejuízos para a resposta à terapia medicamentosa. ^3 , 10 , 11^ Alguns estudos relatam maior incidência de infecções respiratórias ^12^ e o surgimento de novos casos de asma ^3 , 7 , 11^ entre indivíduos que convivem com fumantes.

O Sistema Global de Vigilância ao Tabagismo (GTSS - *Global Tobacco Surveillance System* ) apoia o combate ao fumo, ^13^ colaborando para a redução da prevalência de fumantes em todo o mundo. Contudo, a exposição à fumaça secundária do cigarro ainda é um desafio. ^14^ Segundo dados do Sistema de Vigilância de Fatores de Risco e Proteção para Doenças Crônicas por Inquérito Telefônico (VIGITEL) de 2013, Salvador foi a capital brasileira com menor frequência de exposição à fumaça secundária do cigarro em domicílio (7,5%), ^15^ com queda em 2014 (5,4%). ^16^ Com relação ao ambiente do trabalho, os inquéritos de 2013 e 2014 mostraram um cenário preocupante, com frequência de 9,1% de exposição à fumaça secundária do cigarro. ^15 , 16^ Além de serem frequências mais elevadas do que as do tabagismo ativo (5,1%), ^17^ estes dados preocupam também porque não há métodos eficientes para se evitar a exposição à fumaça do cigarro em ambientes fechados, o que aponta para a necessidade de mecanismos de proteção para quem não fuma. Tendo em vista que se trata de fator de risco modificável, que pode contribuir para a falta de controle da asma, é fundamental conhecer areal situação deste tipo de exposição entre indivíduos asmáticos, com diferentes níveis de gravidade da doença, para que seja possível desenvolver estratégias de intervenção que minimizem seu impacto sobre a doença. ^14^

## OBJETIVO

Estimar a frequência e as características da exposição à fumaça secundária do cigarro em pacientes com asma, e sua associação com o controle e a gravidade da doença.

## MÉTODOS

### Delineamento do estudo

Trata-se de estudo transversal, vinculado à linha de investigação intitulada Fatores de Risco, Biomarcadores e Endofenótipos da Asma Grave, conduzido no Programa para o Controle da Asma na Bahia (ProAR), da Universidade Federal da Bahia, cujos métodos foram previamente descritos. ^18^

### Recrutamento e participantes

Foram elegíveis para este estudo, pacientes com asma grave, asma leve/moderada (ALM) e indivíduos sem asma, adultos (≥18 anos de idade), sem distinção de sexo, residentes em Salvador (BA) ou Região Metropolitana, usuários do Sistema Único de Saúde (SUS). Os participantes com asma grave foram recrutados da coorte do ProAR e selecionados desde que estivessem em acompanhamento regular há pelo menos 6 meses na data da avaliação clínica. ^19^ Todos tiveram seus diagnósticos confirmados no momento da admissão no programa, e a gravidade da doença foi definida considerando-se os critérios da portaria SAS/MS 1.012/2002 e da *Global Initiative for Asthma* (GINA), de 2002. ^19^ Os indivíduos com ALM e sem asma foram recrutados a partir da comunidade, por meio da divulgação de anúncios em unidades de saúde, ambulatórios de especialidades, transportes e outros locais públicos. O diagnóstico de ALM foi confirmado segundo os critérios da GINA. ^20^ Participantes sem asma, identificados após a avaliação clínica, também foram incluídos, para verificação de diferentes padrões de exposição.

Foram excluídos do estudo participantes com comorbidades que impedissem a avaliação precisa do controle da asma, como insuficiência cardíaca congestiva (ICC), miopatias, doença respiratória crônica das vias aéreas inferiores, neoplasia avançada e gravidez. ^18^

Um *call center* foi organizado na sede do ProAR, para informar os interessados sobre detalhes da pesquisa, e checar os critérios de elegibilidade. Pessoas indicadas por pacientes do ProAR também foram contactadas. Para os elegíveis, uma visita era agendada para a assinatura do Termo de Consentimento Livre e Esclarecido (TCLE) e para iniciar a avaliação clínica e demais procedimentos da pesquisa.

### Coleta de dados

O estudo atual avaliou dados de 1.450 indivíduos (996 com asma e 454 AS), coletados entre 2013 e 2015. Questionários padronizados foram aplicados para obter informações sobre características sociodemográficas e de exposição à fumaça secundária do cigarro no ambiente domiciliar, no trabalho/escola e em outros ambientes públicos. Dados sobre tabagismo atual e pregresso, questões sobre o uso de medicações para asma e outras comorbidades, consumo de álcool e de drogas ilícitas também eram colhidas. Em um segundo momento, os entrevistadores aplicavam um questionário adaptado do Instituto Brasileiro de Geografia e Estatística (IBGE) utilizado no Censo 2010. ^21^ O questionário do Ministério da Saúde/VIGITEL também foi utilizado para verificação dos fatores associados ao desenvolvimento de doenças crônicas. ^22^

Adicionalmente, o questionário *Asthma Quality of Life Questionnaire* (AQLQ) foi aplicado para verificar se o indivíduo deixou de frequentar algum ambiente para não se expor à fumaça secundária do cigarro. ^23^

### Avaliação do controle da asma

A avaliação do controle da asma foi realizada por meio do Questionário de Controle da Asma (ACQ), traduzido e validado no Brasil, ^24^ e escores ≥1,5 foram indicativos de asma não controlada.

### Análise estatística

Os dados foram analisados pelo (SPSS), versão18.0. A estatística descritiva e as comparações são apresentadas em tabelas. Para medida de associação, utilizou-se razão de prevalência (RP) e seus respectivos intervalos de confiança de 95% (IC95%).

### Aspectos éticos

Este estudo foi aprovado pelo Comitê de Ética e Pesquisa da Maternidade Climério de Oliveira da Faculdade de Medicina da Universidade Federal da Bahia (pareceres 099/2009 e 095/2012).

## RESULTADOS

### Características sociodemográficas

Apopulação estudada foi composta predominantemente por mulheres (81,8%), com idade entre 36 e 51 anos. Diferenças significativas em relação às variáveis apresentadas foram observadas entre os grupos, exceto em relação à cor autorreferida ( Tabela 1 ).

**Tabela 1:** Caracterização sociodemográfica e autorrelato de tabagismo atual e pregresso de pacientes com asma grave, asma leve e indivíduos sem asma

Característica	Asma grave (n=544)	Asma leve/moderada (n=452)	Sem asma (n=454)	Valor de p	Total (n=1.450)
Sexo feminino	444 (81,6)	351 (77,7)	391 (86,1)	<0,01*	1.186 (81,8)
Idade, anos	51,85±13,47	36,84±12,79	44,07±12,55	<0,01 ^†^	44,74±4,38
Renda familiar, R$	1.186,32±1.272,47	1.412,78±1.073,09	1.290,45±1.458,23	<0,01*	1.287,71±1.284,78
Estado civil				<0,01 ^†^	
Solteiro	195 (35,8)	256 (56,6)	187 (41,2)		638 (44,0)
Casado/união estável	234 (43,0)	163 (36,1)	189 (41,6)		586 (40,4)
Divorciado	59 (10,8)	25 (5,5)	52 (11,5)		136 (9,4)
Viúvo	56 (10,3)	8 (1,8)	26 (5,7)		90 (6,2)
Escolaridade				<0,01*	
Sem instrução	37 (6,8)	3 (0,7)	8 (1,8)		48 (3,3)
Fundamental 1	127 (23,3)	25 (5,5)	51 (11,2)		203 (14,0)
Fundamental 2	129 (23,7)	62 (13,7)	106 (23,3)		297 (20,5)
Médio	215 (39,5)	232 (51,3)	240 (52,9)		687 (47,4)
Superior	36 (6,6)	130 (28,8)	49 (10,8)		215 (14,8)
Cor/raça				0,68*	
Parda	280 (51,5)	214 (47,3)	235 (51,8)		729 (50,3)
Preta	215 (39,5)	195 (43,1)	180 (39,6)		590 (40,7)
Outras ^‡^	49 (9,0)	43 (9,5)	39 (8,6)		131 (9,0)
Tabagismo atual				<0,01*	
Não	539 (99,1)	434 (96,0)	417 (91,9)		1.390 (95,9)
Sim, diariamente	2 (0,4)	8 (1,8)	27 (5,9)		37 (2,5)
Sim, ocasionalmente	3 (0,6)	10 (2,2)	10 (2,2)		23 (1,5)
Tabagismo pregresso				<0,01*	
Não	392 (72,7)	371 (86,1)	326 (77,8)		1.089 (75,1)
Sim	147 (27,3)	60 (13,9)	93 (22,2)		300 (20,7)

Resultados expressos por n (%) ou média±desvio padrão. * teste do χ ^2^ (variáveis categóricas). ^†^ teste de Kruskal-Wallis (variáveis contínuas); ^‡^ branca, indígena e amarela.

### Frequências de tabagismo atual e pregresso

Ao avaliar os dados sobre hábito tabágico, obtidos do questionário do IBGE de 2010 e do VIGITEL, observaram-se menores frequências de tabagismo atual autorreferido entre participantes com asma grave (5 pacientes; 1%), seguidos do grupo com ALM (18;4,0%) e sem asma (37; 8,1%); com p<0,01. Entre os tabagistas atuais, 37 (2,5%) relataram consumo diário de cigarro e 23 (1,6%) consumo ocasional ( Tabela 1 ).

Entre os indivíduos não tabagistas, observou-se o autorrelato de tabagismo pregresso em 300 (20,7%) participantes, sendo 147 (27,3%) com asma grave, 60 (13,9%) com ALM e 93 (22,2%) sem asma ( Tabela 1 ).

### Características de exposição secundária à fumaça do cigarro

Ao serem questionados sobre exposição à fumaça secundária de cigarro no domicílio, 203 (14,0%) participantes informaram que uma ou mais pessoas fumavam em casa, sendo 74 (13,6%) asma grave, 50 (11,1%) ALM e 79 (17,4%) sem asma. Entre os asmáticos graves, 8,2% relataram exposição à fumaça secundária do cigarro no ambiente domiciliar; 19,1% exposição no trabalho/escola e 71,9% exposição diária em ambientes públicos ( Tabela 2 ).

**Tabela 2:** Caracterização da exposição secundária à fumaça do cigarro em diferentes ambientes entre pacientes com asma grave, asma leve a moderada e indivíduos sem asma

		Asma grave (n=544)		Asma leve/moderada (n=452)	Sem asma (n=454)	Valor de p*		Total (n=1.450)	
Quantas pessoas fumam dentro de sua residência?									
Uma	59 (10,8)			38 (8,4)	58 (12,8)		0,07		155 (10,7)
Duas ou mais	15 (2,8)			12 (2,7)	21 (4,6)				48 (3,3)
É permitido fumar em todos os lugares dentro do seu domicílio?									
Sim	43 (7,9)			52 (11,5)	66 (14,5)		<0,01		161 (11,1)
Qual a frequência com que alguém fuma dentro do seu domicílio?									
Diariamente	37 (6,8)			32 (7,1)	48 (10,6)		<0,01		117 (8,1)
Semanal	8 (1,5)			16 (3,5)	3 (0,7)				27 (1,9)
Mensal	5 (0,9)			7 (1,5)	2 (0,4)				14 (1,0)
Menos que uma vez por mês	2 (0,4)			5 (1,1)	16 (3,5)				23 (1,6)
Por quanto tempo você estima estar exposto à fumaça de cigarro em seu domicílio?									
Menos de 1 hora/dia	20 (3,7)			28 (6,2)	29 (6,4)		0,26		77 (5,3)
1 a 4 horas/dia	8 (1,5)			10 (2,2)	10 (2,2)				28 (1,9)
Mais de 4 horas/dia	16 (3,0)			12 (2,7)	19 (4,2)				47 (3,2)
Nas últimas 24 horas, quanto tempo você esteve exposto à fumaça de cigarro?									
Menos de 1 hora/dia	13 (2,4)			13 (2,9)	18 (4,0)		0,38		44 (3,0)
1 a 4 horas/dia	8 (1,5)			10 (2,2)	11 (2,4)				29 (2,0)
Mais de 4 horas/dia	12 (2,2)			29 (1,1)	29 (2,6)				29 (2,0)
Tem atividade extradomiciliar (trabalha ou estuda fora do lar)?									
Sim	242 (44,5)			330 (73,0)	288 (63,4)		<0,01		860 (59,3)
Existem normas sobre fumar em ambientes fechados em seu local de trabalho ou escola?									
Sim	164 (68,0)			234 (71,3)	204 (71,6)		0,61		602 (41,5)
Quantas pessoas fumam perto de você em seu local de trabalho ou escola?									
Uma	20 (8,3)			24 (7,4)	25 (8,8)		0,75		69 (4,8)
Duas ou mais	28 (11,7)			36 (11,1)	40 (14,0)				104 (7,2)
Quantas horas você estima estar exposto à fumaça de tabaco em seu local de trabalho ou escola?									
<1 hora/dia	31 (12,9)			36 (11,0)	27 (9,5)		0,11		94 (6,5)
Entre 1 a 4 horas/dia	3 (1,2)			14 (4,3)	11 (3,9)				28 (1,9)
Mais de 4 horas/dia	12 (5,0)			8 (2,4)	17 (6,0)				37 (2,6)
Nas últimas 24 horas, quanto tempo você esteve exposto à fumaça de tabaco em seu local de trabalho ou escola?									
<1 hora por dia			17 (7,1)	13 (3,9)	21 (7,4)		0,22		51 (3,5)
Entre 1 e 4 horas/dia			2 (0,8)	5 (1,5)	2 (0,7)				9 (0,6)
Mais de 4 horas/dia			7 (2,9)	4 (1,2)	9 (3,2)				20 (1,4)
Com que frequência você está exposto à fumaça de tabaco além do seu domicílio e do trabalho/escola?									
<1 vez/semana	147 (27,6)			163 (36,3)	151 (33,6)		<0,01		461 (31,8)
>1 vez/semana	82 (15,4)			87 (19,4)	80 (17,8)				249 (17,2)
Diariamente	154 (28,9)			131 (29,2)	94 (20,9)				379 (26,1)
Nas últimas 24 horas, quanto tempo você esteve exposto à fumaça do tabaco?									
<1 hora/dia	139 (25,8)			99 (21,9)	91 (20,1)		0,45		329 (22,7)
1 a 4 horas/dia	27 (5,0)			21 (4,6)	27 (6,0)				75 (5,2)
>4 horas/dia	23 (4,3)			19 (4,2)	18 (4,0)				60 (4,1)

Resultados expressos por n (%). * teste do χ ^2^ .

Ao avaliar a exposição no trabalho/escola, observou-se grande proporção de participantes que não exercia atividade extradomiciliar entre os asma grave (55,5%). Entretanto, entre aqueles que trabalhavam ou estudavam, 173 (12,0%) referiam exposição ao tabagismo passivo, devido à presença de um ou mais fumantes em seu ambiente laboral ou escolar.

Não houve diferença significativa entre os grupos, mas chamaram a atenção as elevadas frequências deste tipo de exposição, relatada pelos participantes (20,0% entre asma grave, 18,5% ente ALM e 22,8% entre sem asma). Ademais, 58,5% negaram a existência de normas que impedissem o uso do cigarro no ambiente de trabalho/escola ( Tabela 2 ). Verificou-se também que a exposição à fumaça secundária do cigarro em ambientes públicos foi bastante frequente, sendo referida por 71,9% dos participantes com asma grave, 84,9% entre os que tinham ALM e 72,3% entre os sem asma ( Tabela 2 ).

### Avaliação do controle da asma

Ao analisar os dados sobre a exposição à fumaça secundária de cigarro e controle da asma ACQ, percebeu-se maior ocorrência de asma controlada entre os asmáticos não expostos à fumaça secundária do cigarro quando comparados aos expostos, tanto entre ALM quanto entre asma grave. Entretanto, observou-se associação entre exposição à fumaça secundária do cigarro e controle da asma apenas entre os indivíduos com ALM (RP= 2,04; IC95%: 1,27-3,30) ( Tabela 3 ).

**Tabela 3:** Associação entre exposição à fumaça secundária do cigarro e controle da asma, segundo a gravidade da doença

Exposição	Asma grave (n=544)			Asma leve/moderada (n=452)		
	
	Controlado (n=352)	Não controlado (n=192)	RP (IC95%)	Controlado (n=386)	Não controlado (n=66)	RP (IC95%)
Presente	56 (15,9)	33 (17,2)*	0,91	52 (13,5)	18 (27,3)*	2,04
			(0,57-1,46)			(1,27;3,30)
Ausente	296 (84,1)	159 (82,8)*	p=0,7	334 (86,5)	48 (72,7)*	p<0,01

Resultados expressos por n (%).* Questionário de Controle da Asma ≥1,5 (asma não controlada).

RP: razão de prevalência; IC95%: intervalo de confiança de 95%.

Ao avaliar a proporção de indivíduos com asma que evitaram frequentar determinados ambientes pelo receio da exposição à fumaça secundária do cigarro, de acordo com os dados do AQLQ, observou-se que a maioria dos pacientes com asma tinha sua vida social limitada, pelo receio dessa exposição. Indivíduos com asma grave referiram evitar ambientes por este motivo 18% mais que os com ALM (RP= 1,18; IC95%: 1,04-1,35) ( Tabela 4 ).

**Tabela 4:** Proporção de indivíduos com asma leve, moderada e grave que evitaram estar presentes em ambientes por receio de exposição à fumaça secundária do cigarro

	Asma grave (n=544)	Asma leve/moderada (n=452)
Evitaram	386 (71,0)	285 (63,1)
Não evitaram	158 (29,0)	167 (36,9)

Resultados expressos por n (%). Razão de prevalência (intervalo de confiança de 95%) =1,18 (1,04-1,35).

## DISCUSSÃO

Este estudo trouxe informações relevantes sobre tabagismo secundário ou passivo, que, embora seja conhecido de longa data, permanece como situação de difícil abordagem. Os resultados demonstraram que, entre os participantes com asma grave, os relatos de tabagismo pregresso e exposição à fumaça secundária do cigarro foram frequentes. Esse grupo também apresentou maior proporção de indivíduos que desconhecem as normas regulamentadoras do fumo em ambientes fechados e maior exposição à fumaça secundária do cigarro em ambientes públicos. O estudo ainda verificou maior frequência de asma não controlada entre os expostos à fumaça secundária do cigarro, especialmente no grupo com ALM, e mais relatos de deixarem de frequentar alguns locais para evitar a exposição entre os pacientes com asma grave.

Quanto às características sociodemográficas, no grupo de participantes com asma grave, houve maior proporção de mulheres e de participantes que não trabalhavam/estudavam, média de idade mais elevada e baixo nível de escolaridade. Este perfil também foi observado em estudo semelhante. ^5^

Foi maior a proporção de indivíduos que não exercia atividade laboral entre os asmáticos graves, o que pode refletir maior gravidade e falta de controle dos sintomas e mais visitas às unidades de emergência e hospitalizações, contribuindo para o absenteísmo laboral/escolar. ^5 , 25^

A baixa escolaridade também foi observada por outros autores. ^26^ Segundo o último censo demográfico brasileiro, 50,2% da população com mais de 10 anos de idade nunca estudou ou possui apenas o Ensino Fundamental incompleto, justificando o perfil dos participantes deste e de outros estudos semelhantes. ^27^ O grau de instrução deve ser considerado no planejamento de ações de educação em saúde, pois pode interferir na compreensão dos instrumentos e da intervenção por parte dos participantes, podendo representar um viés de coleta dos dados.

Este estudo encontrou menor frequência de tabagismo atual entre asmáticos, que pode ter ocorrido por limitação do método empregado (autorrelato). ^3 , 10^ Estudo realizado em São Paulo também encontrou baixa frequência de tabagistas atuais (3%) e passivo (17%) em população de asmáticos, e os autores discutem a importância de se utilizarem técnicas objetivas para detecção de biomarcadores do tabagismo, como a medida da cotinina urinária. ^14^ Estudo realizado por nosso grupo, com o objetivo de comparar o tabagismo ativo identificado por autorrelato e valores de cotinina urinária, revelou taxa de omissão de 5% entre asma grave e 4,8% entre pacientes com ALM, indicando a importância do uso de medidas objetivas, nos casos em que o monitoramento do tabagismo for importante para o manejo da doença. ^28^

A exposição à fumaça secundária do cigarro foi relatada por parte significativa dos participantes do atual estudo, asmáticos e sem asma. Apesar da frequência de tabagismo atual ter sido relativamente baixa entre os asmáticos, observou-se que eles continuavam expostos à fumaça secundária do cigarro no ambiente domiciliar, mesmo aqueles com doença grave. Entretanto, foram os indivíduos sem asma os que mais relataram a presença de fumantes no ambiente domiciliar, a liberdade para uso do cigarro em qualquer local dentro do domicílio e a exposição à sua fumaça em casa. Embora esses indivíduos talvez não experimentem desconforto imediato relacionado à exposição à fumaça do cigarro, dados de diferentes estudos revelam maior risco de desenvolver asma para quem convive com indivíduos que fumam. ^3 , 7 , 11^ Esse fato deve ser lembrado nos aconselhamentos em saúde, tanto de quem fuma quanto de quem convive com fumantes. Tal abordagem torna-se ainda mais importante no manejo da asma, já que a exposição à fumaça secundária do cigarro pode estar relacionada, entre outros fatores, à falta de resposta ao tratamento. ^3^

A permissão para fumar em qualquer lugar da residência, a despeito da gravidade da doença, foi relatada por quase 10% dos asma grave e 13,3% dos participantes com ALM. Este achado sugere desinformação por parte dos familiares e/ou do próprio paciente, reforçando a importância das ações de educação em saúde no acompanhamento do paciente com asma. Informar sobre os riscos faz parte do processo de empoderamento do paciente e de seus familiares, levando-os a se corresponsabilizem pelo tratamento e pela melhora clínica. O tabagismo atual e a exposição secundária à fumaça do cigarro contribuem para a redução da resposta terapêutica aos corticosteroides e ao pior controle da asma, ^3 , 29^ além de contribuir para o declínio da função pulmonar, mais acentuado entre asmáticos tabagistas do que entre asmáticos não tabagistas, ^30^ fato que pode ser observado também em filhos de pais fumantes. ^31^

A lei federal 12.546/2011, proibindo o tabagismo em locais fechados em todo o país, ^32^ e o decreto presidencial 8.262/2014 ^33^ regulamentam medidas para promover ambientes livres do cigarro. Apesar disso, 30% dos indivíduos que trabalham/estudam referiram desconhecer normas que proíbem o tabagismo em ambientes fechados, levantando a questão sobre a necessidade de divulgação dessas informações e da implantação de ações de vigilância.

Estudo realizado entre escolares encontrou prevalência elevada de indivíduos expostos à fumaça secundária do cigarro em ambientes públicos (62,2% em Porto Alegre e 53,6% em Florianópolis). ^31^ Estes dados são semelhantes aos observados neste estudo, que identificou frequência diária de exposição à fumaça secundária do cigarro em ambientes públicos, admitida por 28,9% dos pacientes com asma grave e 29,0% dos com ALM. A falta de restrição ao tabagismo em ambientes públicos pode afetar os que apresentam doenças respiratórias crônicas, ^22^ o que reforça a necessidade de ações públicas eficazes para restringir essa exposição, mesmo em ambientes abertos. ^16^

O atual estudo identificou exposição à fumaça secundária do cigarro nas últimas 24 horas em proporção considerável de participantes, especialmente entre os com asma grave, indicando contato frequente e atual. É possível que os asmáticos sejam mais sensíveis e, portanto, consigam perceber melhor a presença de fumaça no ambiente e tentem mais frequentemente evitá-la; porém os métodos do atual estudo não permitiram esta análise.

Ao avaliar a relação entre a exposição à fumaça secundária do cigarro e o controle da asma, encontrou-se que pacientes com ALM apresentam 2,04 vezes mais chances de apresentar asma não controlada ao contato com a fumaça secundária do cigarro do que pacientes com asma grave, dado que sugere que a falta de controle dos sintomas entre os pacientes com asma grave pode estar ligada a características biológicas da doença, estando pouco sujeita a fatores externos, o que pode não acontecer na ALM. Vários estudos mostram associação entre a exposição e piora da inflamação das vias aéreas e da HRB, aumento do número de exacerbações, prejuízo da qualidade de vida, falta de controle e maior gravidade da doença. ^3 , 10 , 11^ King et al., realizando estudo de corte transversal em 14 países, encontraram frequência significativamente elevada de exposição à fumaca secundária do cigarro em diversos ambientes e discutiram a necessidade intervenções profissionais. ^34^ Outro estudo mostrou que o tabagismo interfere significativamente no aumento das doses de corticosteroides inalatórios utilizados pelos asmáticos e no controle da doença. ^35^ A proporção de indivíduos com asma expostos à fumaça secundária do cigarro no presente estudo foi considerável, o que deve alertar os profissionais que tratam asma sobrea importância de orientar os pacientes a evitarem este tipo de exposição, e que solicitem aos fumantes ativos próximos que se abstenham de fumar em sua presença, ^3 , 14^ pelo risco de terem seus sintomas exacerbados e a doença descontrolada.

As consequências dessa exposição para a vida dos pacientes com asma vão além das repercussões clínicas. A qualidade de vida dessas pessoas é afetada não apenas nos períodos de exacerbação, quando precisam de mais visitas à emergência e hospitalizações. Quando as respostas ao questionário AQLQ foram analisadas, observou-se que aproximadamente 68% do total de asmáticos estudados admitiram não frequentar certos ambientes com receio de provável exposição à fumaça secundária do cigarro, e esta atitude foi significativamente mais relatada pelos pacientes com asma grave. Isto revela que estas pessoas também são afetadas no dia a dia, mesmo nos períodos de melhor controle da doença, pois, para evitaras consequências da exposição à fumaça secundária do cigarro, os pacientes têm sua mobilidade restrita.

## CONCLUSÃO

Este estudo identificou que um terço dos asmáticos (tanto com asma leve/moderada quanto asma grave) é frequentemente expostos à fumaça secundária de cigarro no domicílio, no ambiente de trabalho/escola e/ou ambientes públicos. Mais de dois terços dos pacientes com asma grave relataram evitar ambientes com maiores chances de exposição, por receio das exacerbações e suas consequências, o que é inaceitável. Esta exposição também está associada à falta de controle da doença entre os pacientes com asma leve/moderada. Novos estudos sobre esse tema devem ser incentivados, para a confirmação destes resultados em outras regiões e situações. Estas informações devem inspirar novas intervenções destinadas à prática clínica diária e a criação de politicas públicas, visando ao benefício dos pacientes com asma.
